# Emergency department diagnosis of pulmonary hypertension in a patient with left atrial sarcoma

**DOI:** 10.1186/s12245-014-0032-5

**Published:** 2014-09-30

**Authors:** Maricel Dela Cruz, Jeremy Seelinger Devey

**Affiliations:** 1 Aria Health Philadelphia, Philadelphia 19124, PA, USA; 2 Gallup Indian Medical Center, Gallup 87301, NM, USA

## Abstract

Pulmonary hypertension is a disease with many etiologies and is responsible for 200,000 admissions and 25,000 hospitalizations in the United States each year. We report the case of a previously healthy 58-year-old woman who presented to the emergency department with a months-long history of worsening dyspnea on exertion, orthopnea, and paroxysmal nocturnal dyspnea. Despite the severity of her symptoms, she had no corroborative physical exam findings, including jugular venous distension or peripheral edema. Bedside emergency department ultrasonography revealed a dilated right ventricle and bowing of the intraventricular septum into the left ventricle, consistent with pulmonary hypertension. CT angiography of the chest performed in the emergency department revealed a large left atrial mass, found on pathology to be a left atrial sarcoma. This case illustrates how severely symptomatic pulmonary hypertension can have few to no physical exam findings and the utility of bedside emergency department ultrasound in making the presumptive diagnosis.

## 1 Background

Approximately 200,000 hospitalizations occur annually in the United States due to pulmonary hypertension (PH) [1]. Although the exact prevalence of pulmonary hypertension is unknown, its significance is noteworthy as it is directly responsible for 25,000 deaths per year [1]. The causes of pulmonary hypertension are numerous with the majority of emergency department presentations attributed to heart failure and pulmonary thromboembolism [2]–[8].

We present a case report of a patient with progressively worsening orthopnea and dyspnea on exertion without clinical signs of congestive heart failure. The patient exhibited characteristics of pulmonary hypertension on bedside emergency department echocardiogram and was subsequently found to have a left atrial mass on computed tomography angiogram of the chest.

## 2 Case presentation

A 58-year-old Native American female presented to the emergency department with a 3-month history of progressive shortness of breath, orthopnea, and dyspnea upon exertion. Initially, the patient experienced only mild exercise intolerance but by the time of her presentation to the emergency department (ED), she noted that she was becoming winded after only a few steps. The patient's orthopnea had become so intolerable that she now slept upright in a recliner every night. Associated symptoms included paroxysmal nocturnal dyspnea and a non-productive cough. The patient denied chest pain, lower extremity edema, weight loss, or episodes of syncope. The patient had seen her primary care physician several times for these symptoms. Her doctor had performed a chest x-ray, diagnosed bronchitis, and arranged a sleep study that had not yet been performed. The patient's only known medical problem was gastroesophageal reflux disease for which she was taking omeprazole. The patient had no surgical history, medication allergies, and drank no alcohol and did not smoke or use illicit substances.

**Figure 1:**
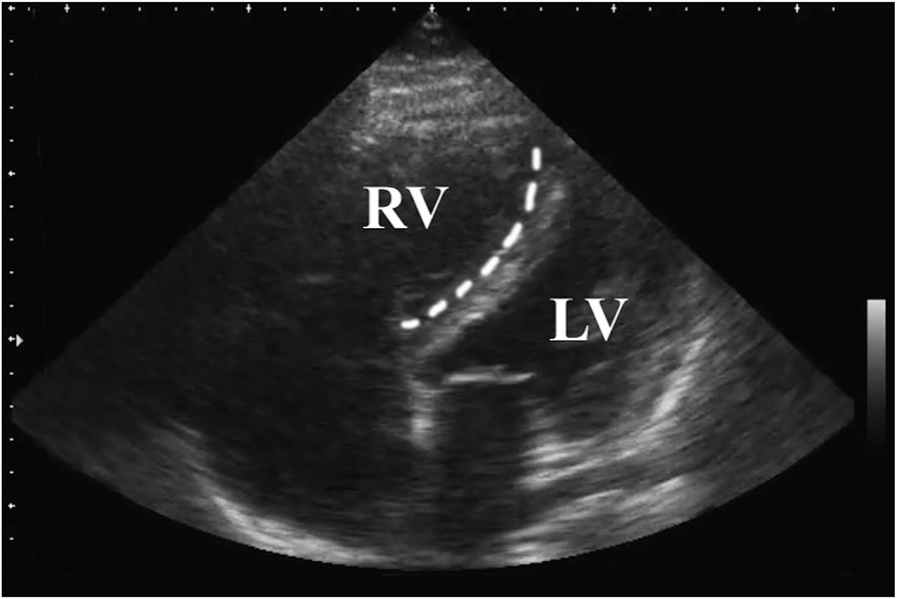
**Representative image of pulmonary hypertension by bedside echocardiogram.** The right ventricle (RV) is dilated and the intraventricular septum (dashed line) is bowing into the left ventricle (LV). (Courtesy of Geoffrey Hayden, MD).

Upon examination, the patient was not in acute distress. Vital signs including blood pressure, temperature, heart rate, respiratory rate, and pulse oximetry were all within normal limits. The cardiovascular exam demonstrated a regular rate and rhythm with no audible murmurs, gallops, or rubs. There was no jugular venous distension. There was trace lower extremity edema at the right pretibial region. Lung sounds were clear bilaterally, and there was no respiratory distress. The remainder of the physical exam was normal. Bedside echocardiogram showed a grossly dilated right ventricle, non-collapsibility of the right ventricle throughout the cardiac cycle, and bowing of the intraventricular septum into the left ventricle (Figure 1).

**Figure 2:**
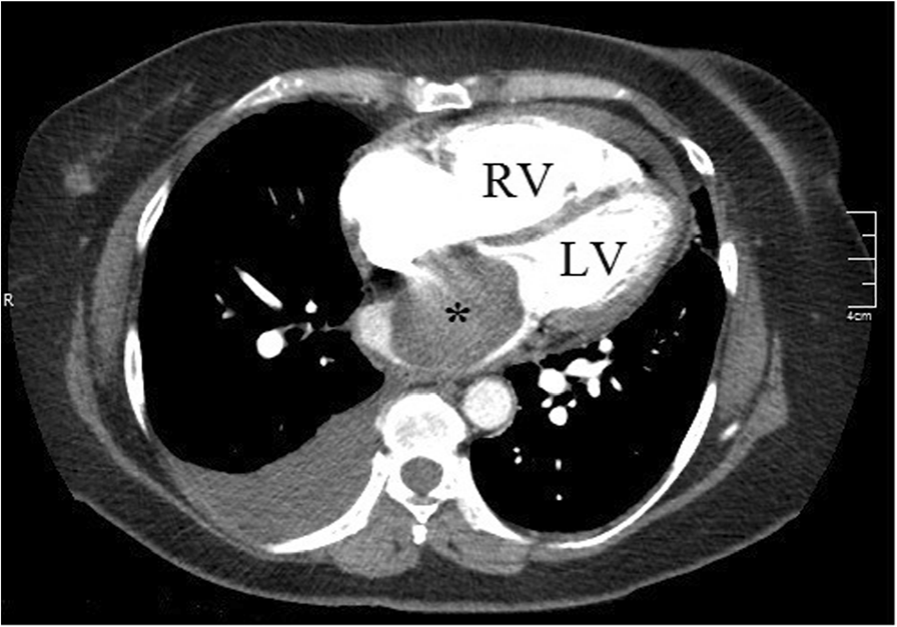
**Computed tomography angiogram of the chest exhibiting a left atrial filling defect (asterisk).** Note the size of the RV in relation to the LV.

Laboratory workup included a metabolic panel and a complete blood count that were unremarkable, as well as a D-dimer which was elevated at 1,345 ng/ml. The patient's electrocardiogram revealed T wave inversions in leads V_1_ through V_3_ with extreme axis deviation. Chest x-ray demonstrated a small right pleural effusion. Computed tomography angiogram (CTA) of the chest was ordered to rule out pulmonary embolism (Figure 2). CTA was negative for pulmonary embolism but did demonstrate a 6.3 × 4.3 cm filling defect of the entire left atrium that was suspicious for cardiac tumor. A right pleural effusion was also confirmed.

## 3 Discussion

As with many conditions, PH has both primary and secondary causes. Primary PH can be either familial or idiopathic. Secondary PH causes include pulmonary thromboembolic disease, congestive heart failure, COPD, obstructive sleep apnea, pulmonary fibrosis, connective tissue disease, congenital heart disease, cardiomyopathy, portal hypertension, certain toxins, human immunodeficiency virus, and other infectious causes [1]. Primary cardiac tumors, such as that responsible for our patient's PH, are extremely rare often presenting with symptoms that can mimic more common diseases that are regularly encountered in the emergency department [1],[9]–[12].

Long-term survival in patients with pulmonary hypertension varies according to its etiology. Five-year survival for congenital heart disease patients with PH approaches 80%, while HIV patients with PH demonstrate only 20% survival at 3 years [13].

Exertional dyspnea is the most common symptom attributable to pulmonary hypertension [1]. Other common symptoms include fatigue, chest pain, syncope, and peripheral edema. Physical exam findings may include increased jugular venous pressure, palpable right ventricular impulse, increased second heart sound, and an S4. Peripheral cyanosis and edema are late findings [1]. Physical exam may be normal or near-normal in the early stages of disease. Chest x-ray can sometimes display enlarged central pulmonary arteries, increased distal pulmonary vasculature markings, and/or right atrial and right ventricular enlargement, though these findings are poorly sensitive [1]. Electrocardiogram may reveal right axis deviation, right ventricular hypertrophy, and right atrial enlargement [1],[6].

Traditionally, a diagnosis of PH could only be suspected by the emergency physician, as confirmatory testing was not possible from the ED. In the age of bedside ED ultrasound, however, the emergency physician can make a presumptive diagnosis of PH on the basis of a few simple parameters. Echocardiogram is one of the most useful tests in the diagnosis of pulmonary hypertension [1],[14],[15] usually revealing right ventricular enlargement, elevated pulmonary artery pressures as calculated by Doppler flow across the tricuspid valve, and abnormalities of the interventricular septum, including bowing of the septum into the left ventricle and paradoxical movement of the septum into the left ventricle during systole. Later stages of pulmonary hypertension may lead to findings of tricuspid regurgitation, right ventricular hypertrophy, and right ventricular hypokinesis [1],[14]–[16]. One cause of PH, acute pulmonary embolism, is associated with a fairly specific echocardiographic finding: McConnell's sign. Patients with McConnell's sign will demonstrate akinesis of the mid free wall of the right ventricle with sparing of the apex [17]. The emergency physician using bedside ED ultrasonography can suspect PH of any cause when the right ventricle, normally only 60% of the size of the left, appears dilated and when abnormalities of the intraventricular septum, such as bowing and paradoxical movement, are present.

The treatment of PH in the emergency department is focused on the underlying etiology and may include afterload reduction for congestive heart failure, emergent thrombolysis for pulmonary embolism, or admission for further evaluation and medical treatment of other forms of pulmonary hypertension. For primary cardiac tumors causing PH, surgical resection and, when appropriate, adjuvant chemotherapy have been shown to be the treatments with the best long-term outcomes [9],[10],[12],[18]–[20].

## 4 Conclusion

The patient was placed on oxygen therapy and admitted to the intensive care unit for close monitoring. She was evaluated by a cardiologist who confirmed PH by formal echocardiography and recommended transfer to a cardiology specialty hospital. The patient was transferred and subsequently taken to the operating room for resection of the left atrial mass. Pathology findings from the mass were consistent with sarcoma, a malignant primary cardiac tumor with an incidence of 0.0001% [21]. The patient underwent post-operative chemotherapy and several months after the surgery had experienced significant improvement in her symptoms.

### 4.1 Key point

Emergency department recognition of pulmonary hypertension may be expedited by the use of bedside ultrasound and can help direct physicians towards further workup, management, and definitive treatment.

## Abbreviations

CTA: computed tomography angiogram

COPD: chronic obstructive pulmonary disease

ED: emergency department

HIV: human immunodeficiency virus

PH: pulmonary hypertension

## Competing interests

The authors declare that they have no competing interests.

## Authors' contributions

MDC performed literature review and drafted the manuscript. JSD conceived of, researched, edited, and revised the manuscript and obtained the relevant images. Both authors read and approved the final manuscript.

## Authors' information

MDC is a resident in the combined family medicine/emergency medicine residency program at Aria Health in Philadelphia, PA, USA. JSD is an emergency physician at Gallup Indian Medical Center in Gallup, NM, USA.
